# Effects of Nickel Treatment on H3K4 Trimethylation and Gene Expression

**DOI:** 10.1371/journal.pone.0017728

**Published:** 2011-03-24

**Authors:** Kam-Meng Tchou-Wong, Kathrin Kiok, Zuojian Tang, Thomas Kluz, Adriana Arita, Phillip R. Smith, Stuart Brown, Max Costa

**Affiliations:** 1 Department of Environmental Medicine, New York University School of Medicine, Tuxedo, New York, United States of America; 2 Department of Cell Biology, New York University School of Medicine, Tuxedo, New York, United States of America; 3 Center for Health Informatics and Bioinformatics, New York University School of Medicine, Tuxedo, New York, United States of America; Florida State University, United States of America

## Abstract

Occupational exposure to nickel compounds has been associated with lung and nasal cancers. We have previously shown that exposure of the human lung adenocarcinoma A549 cells to NiCl_2_ for 24 hr significantly increased global levels of trimethylated H3K4 (H3K4me3), a transcriptional activating mark that maps to the promoters of transcribed genes. To further understand the potential epigenetic mechanism(s) underlying nickel carcinogenesis, we performed genome-wide mapping of H3K4me3 by chromatin immunoprecipitation and direct genome sequencing (ChIP-seq) and correlated with transcriptome genome-wide mapping of RNA transcripts by massive parallel sequencing of cDNA (RNA-seq). The effect of NiCl_2_ treatment on H3K4me3 peaks within 5,000 bp of transcription start sites (TSSs) on a set of genes highly induced by nickel in both A549 cells and human peripheral blood mononuclear cells were analyzed. Nickel exposure increased the level of H3K4 trimethylation in both the promoters and coding regions of several genes including CA9 and NDRG1 that were increased in expression in A549 cells. We have also compared the extent of the H3K4 trimethylation in the absence and presence of formaldehyde crosslinking and observed that crosslinking of chromatin was required to observe H3K4 trimethylation in the coding regions immediately downstream of TSSs of some nickel-induced genes including ADM and IGFBP3. This is the first genome-wide mapping of trimethylated H3K4 in the promoter and coding regions of genes induced after exposure to NiCl_2_. This study may provide insights into the epigenetic mechanism(s) underlying the carcinogenicity of nickel compounds.

## Introduction

The nucleosome, the fundamental subunit of chromatin, is composed of 146 bp of DNA wrapped around an octamer of four core histone proteins (H3, H4, H2A, and H2B) [1]–[3]. Post-translational modifications (i.e., acetylation, methylation, phosphorylation, and ubiquitination) of the N- and C-terminal tails of the four core histones play an important role in regulating chromatin biology [4]. These specific histone modifications and their combinations, are translated, through protein interactions, into distinct effects on nuclear processes, such as activation or inhibition of transcription [5]. The implementation and removal of these post-translational modifications has been shown to be fundamental to the regulation of diverse biological processes such as DNA replication, repair and transcription [6].

In eukaryotes, methylation of lysine 4 in histone H3 (H3K4) in the promoter region of genes has been linked to transcriptional activation [7]–[9]. H3K4, as well as other lysines in the tails of histone proteins that are subject to methylation, can occur in mono-, di-, and tri-methylated forms [8], [10], [11]. High levels of H3K4 trimethylation are associated with the 5' regions of active genes. There is a strong positive correlation between trimethylation of H3K4, transcription rates, active polymerase II occupancy and histone acetylation [12]. However, the patterns of H3K4 methylation differ between yeast and vertebrate chromatin. In *Saccharomyces cerevisiae*, dimethylated H3K4 appears to be spread throughout the genes, peaking toward the middle of the coding region, and is associated with transcriptional “poised” as well as active state while monomethylation, was found to be most abundant at the 3′ end of genes [12]. In vertebrates, the majority of dimethylated H3K4 colocalizes with trimethylated H3K4 in discrete zones proximal to highly transcribed genes. Interestingly, there exists a subset of dimethylated sites in the absence of trimethylated H3K4 and these regions do not correlate with transcriptional start sites but instead their presence is highly dependent on the cell type tested [12]. In human T lymphocytes, although all three states of H3K4 methylation were elevated surrounding the transcription start sites (TSSs) of known genes, high levels of H3K4me3 were correlated with highly expressed genes [13].

Occupational exposures to nickel compounds have been implicated in the etiology of human lung and nasal cancers [14]. Nickel compounds have also been shown to activate hypoxia signaling pathways by blocking iron uptake levels leading to cellular iron depletion directly inhibiting prolyl hydroxylase that targets HIF-1 alpha for degradation. Over the past few years, epigenetic mechanisms have been implicated in the carcinogenic actions of nickel compounds. Previous studies have shown that *in vitro* exposure of to nickel results in intracellular accumulation of nickel ions, changes in DNA methylation patterns, and histone modification levels [15]–[18]. These changes can result in alterations in gene expression, thus providing a plausible non-genotoxic mechanism to any heritable alterations in gene expression induced by nickel exposure.

We have shown that exposure of the human lung adenocarcinoma A549 cells to nickel compounds increased global H3K4 trimethylation levels [19]. Because trimethylation of H3K4 is a transcriptional activating mark that defines the location of the promoter region of genes and RNA transcripts [20]–[22], we investigated whether nickel exposure effects levels of trimethylated H3K4 in genes whose expression are highly induced by this metal in the A549 human lung adenocarcinoma cell line. The A549 cell line has been well established as a model for studying the toxic and epigenetic effects of metals since these human lung cells are relevant to the airborne route of exposure of nickel containing compounds [17], [19]. However, since A549 cells are already transformed, we acknowledge that some gene changes may be missed by using A549 cells instead of normal lung cells.

## Materials and Methods

### Cell Culture and Treatment

A549 cells were cultured in F-12K medium (Invitrogen, Carlsbad, CA) at 37°C in an incubator with a humidified atmosphere containing 5% CO_2_. All medium was supplemented with 10% fetal bovine serum (FBS, ATLAS Biological, Fort Collins, CO) and 1% penicillin/streptomycin (Invitrogen CA). Cells were passaged at 80–90% confluence by trypsinization. All treatments were administered when cell density reached approximately 70–80% confluence. A549 cells were treated with 1 mM nickel chloride (NiCl_2_) (Sigma, St. Louis, MO) for 24 hr. Peripheral blood mononuclear cells (PBMCs) were isolated from blood by Ficoll-Hypaque and treated with 0.5 mM NiCl_2_ for 24 hr. Cells were harvested with or without fixation with formaldehyde to crosslink DNA to associated proteins. To crosslink A549 cells and PBMCs, formaldehyde was added directly to the medium to a final concentration of 1% for 10 min and 0.7% for 5 min, respectively.

### Chromatin Immunoprecipitation (ChIP) Assay

ChIP assay was performed according to CHIP protocol from Affymetrix Inc. (Santa Clara, CA). In brief, formaldehyde-crosslinked A549 cells (2.5×10^7^) and PBMCs (1.5×10^7^) were washed three times with lysis buffer, resuspended in pre-IP dilution buffer at 2.0×10^7^ and 1.25×10^7^ cells/ml, respectively, and sonicated at maximum power for 30 minutes (30 pulses, 30 seconds intervals) using Diagenode Bioruptor, equipped with 15 ml tube adaptors. The sonicated and or non-sonicated (non-crosslinked) chromatins were digested with micrococcal nuclease (NEB, Ipswich, MA) at 3333 units nuclease/ml of chromatin at 37C. The digestion time for the crosslinked and non-crosslinked A549 chromatins was 10 minutes with 0.8 and 2.0 mM CaCl_2_, respectively, and 15 minutes for PBMC chromatin supplemented with 2.5 mM CaCl_2_. Reactions were stopped by addition of 5 mM EGTA and 10 mM EDTA. Anti-trimethyl-histone H3 (Lys4) clone MC315 (Millipore Corp. Billerica, MA) monoclonal antibody was used for each ChIP reaction.

### Library Preparation and ChIP-Sequencing (ChIP-Seq)

Five nanograms of the immunoprecipitated DNA was used for the ChIP-Seq DNA library preparation. Immunoprecipitated DNA was end-repaired using End-It DNA repair kit (Epicentre, Madison, WI), followed by the addition of “A” base to the 3′ ends by Klenow with 3′to5′ exonuclease (NEB, Ipswich MA) and the ligation of the Illumina's adapters to the DNA fragments by LigaFast DNA Ligase (Promega, Madison, WI). One or two microliters of the modified DNA were used for Phusion High-Fidelity PCR (NEB). The sequences of the two PAGE purified primers were: 5′CAAGCAGAAGACGGCATACGAGCTCTTCCGATCT-3′ and 5′AATGATACGGCGACCACCGAGATCTACACTCTTTCCCTACACGACGCTCTTCCGATCT-3′ (Invitrogen, CA). Amplified and purified DNA was separated on a 1.8% TAE agarose (Invitrogen, CA) at 115 Volts for 45 minutes. DNA in size ranging from 175–450 bp was excised and purified from the agarose using Qiagen Gel Extraction Kit. DNA concentration was adjusted to 10 nM based on PicoGreen measurements before it was sent out for sequencing.

We performed cluster amplification and 36-nucleotide single-end sequencing using the Illumina Genome Analyzer II (GAII) following manufacturer's protocols. Raw images were analyzed by Illumina RTA (versions 1.4 and 1.6) using Phix control lane for estimating base calling parameters. Pipeline versions 1.4 and CASAVA version 1.6 were used to generate sequence reads, remove first and last bases, and align reads to the human reference genome version NCBI36/hg18. Genome alignments were made with the Illumina ELAND algorithm which uses the first 32 bases of each read as a seed. Post-filters were applied to Illumina's alignment output. Reads that align equally well to multiple positions on the genome were removed and duplicated reads were removed in order to minimize PCR amplification bias.

### RNA Isolation and RNA-Sequencing (RNA-Seq)

Total RNA was isolated from untreated or nickel-treated cells and polyA^+^ RNA was purified using poly-T oligo-attached magnetic beads in accordance to Illumina's protocol for sequencing of mRNA (RNA-Seq). Following purification, the mRNA was fragmented into small pieces using divalent cations under elevated temperature. Then the cleaved RNA fragments were copied into first strand cDNA using reverse transcription and random primers, and followed by second strand cDNA synthesis using DNA Polymerase I and RNaseH. These cDNA fragments were then end repaired, and a single ′A′ base was added to allow blunt-end ligation of adapters. These products were then purified and enriched with PCR to create the final cDNA library suitable for high throughput DNA sequencing on the Illumina Cluster Station and Genome Analyzer.

For RNA-Seq sequencing, each mRNA sample was uploaded onto one lane of flow cell and sequenced in 36-nucleotide single-end run by Illumina Genome Analyzer II (GAII). Raw images were analyzed by Illumina RTA version 1.4 using Phix control lane for estimating base calling parameters. Reads were generated and aligned to human genome version NCBI36/hg18 and exon-exon splice junction database by Illumina CASAVA version 1.5 using default filtering parameters. Exon-exon splice junction database was prepared using UCSC annotation database (January 2010). The last base was trimmed out.

### Analysis of ChIP-Seq Data

For each sample, about 20 million raw reads were generated by ChIP-Seq using the Illumina GAII (Table 1). 85–90% of the reads aligned to the human genome (NCBI36/hg18) using Illumina CASAVA software with default ELAND alignment parameters. After removal of poor quality, multiple alignments, and duplicated reads, 30–40% of reads remained. Due to the differences in the number of reads between experimental data and input DNA data, a random sub-selection was applied to the larger data set for normalization.

**Table 1:** Information of ChIP-Seq Data Sets from crosslinked and non-crosslinked chromatins isolated from control or nickel-treated A549 cells.

	H3K4me3 (Crosslinked Chromatin)			H3K4me3 (Non-Crosslinked Chromatin)		
Number of reads/peaks	Control	Nickel-treated	Input DNA	Control	Nickel-treated	Input DNA
Total	20593983	20896340	19663267	22062122	17966887	25076112
Aligned	18071835	18136592	17512409	18746797	16190322	21683079
Filtered	8352617	7005853	5317783	8263602	8385324	11967312
Used for identifying peaks	5345675	5324449	5317783	8263602	8385324	8377119
Peaks found	14120	14858		23427	25116	
Combined	14762			25778		

The MACS program [23] and locally developed software (TRLocator) were used to identify qualified peak regions in both experimental data and input DNA data with a consolidating window size of 100 bp, p-value<0.05, log2-ratio of enrichment between experimental data and control data > = 1.0, and a minimum tag threshold per peak of 40. Overall false discovery rates (FDR) for all peak finding data sets are less than 1%. To compare nickel-treated versus control H3K4me3 ChIP data sets, the two lists of peaks were matched based on overlapped genome positions. Matched peak regions were annotated using UCSC annotation database (January 2010) and genes with qualified peaks within 5,000 bp of the Transcriptional Start Site (TSS) were identified. Fold-change was also calculated for each matched peak region comparing nickel-treated versus control H3K4me3 peaks. The annotated peak regions were also matched to RNA-Seq gene expression values based on gene name.

### Analysis of RNA-Seq Data

18.6 million (18.6 M) reads each for control and nickel-treated samples were produced from one lane of Illumina GAII. About 56% of reads (10.4 M) for control and 54% (10.1 M) for nickel-treated have alignments mapped to the reference genome after filtering for repeats and reads aligned to multiple genes. Both raw and normalized [base pairs per kilobase per million reads of mapped reads (RPKM)] [24] gene counts were obtained using CASAVA version 1.6 and Genome Studio version 1.6. Fold-change of normalized counts between nickel-treated and control samples were calculated. Out of 21,965 unique genes from the UCSC database, 73% of genes have at least one read aligned (16,067 genes for control and 16,171 genes for nickel-treated). 66% (14,540) of genes have at least one read aligned for both control and nickel-treated samples.

### Combination of ChIP-Seq and RNA-Seq

The set of 10,634 RNA-Seq genes from nickel-treated A549 cells with RPKM greater than 1.5 (about 73% out of 14,540 genes) was matched to the set of genes with qualified H3K4me3 peaks within 5,000 bp flanking TSSs as identified from the ChIP-Seq dataset. Global H3K4me3 profiling curves were generated for all genes identified from the latter to plot the read distributions of H3K4me3 within 5,000 bp upstream and downstream of TSSs. In addition, H3K4me3 profiling curves were also generated for the top 50 highest nickel-induced genes (Table 2). For each of these sets of genes (global or top 50), the tags were binned into 100 bp bins for each gene within 5,000 bp flanking both sides of the TSSs. The number of base pairs within each bin was plotted against the bin distance to TSS. The H3K4me3 profiling curves of four of the top 50 highest nickel-induced genes, namely, CA9, NDRG1, ADM and IGFBP3 were also plotted.

**Table 2:** A list of the top 50 highest nickel-induced genes with qualified H3K4me3 peaks within 5,000 bp flanking the TSSs from crosslinked and non-crosslinked chromatins isolated from nickel-treated and control A549 cells and the fold induction in gene expression determined by RNA-Seq between nickel-treated and control A549 cells was listed.

Top 50 Nickel-Induced Genes	Fold ChangeH3K4me3 Peaks (Crosslinked)	Fold Change H3K4me3 Peaks (Non-Crosslinked)	Fold Induction inGene Expression (RNA-Seq)
CA9	11.65	5.80	54.64
NDRG1	3.92	1.74	24.60
TMEM45A	0.72	1.11	23.87
ANGPTL4	15.67	0.54	20.68
ARRDC4	1.74	3.67	20.08
C15orf48	1.71	1.47	18.53
EGLN3	10.00	113.00	17.21
DNAJB13	67.00	20.20	16.99
RIMKLA*	1.90	-	16.33
CCNG2	1.47	1.01	16.00
WISP2	11.00	6.21	15.31
PLIN2	3.18	40.00	14.97
IGFBP1	2.32	2.61	14.87
EDN2	4.50	4.09	13.59
PDK1	1.35	1.31	13.56
LOX	1.04	1.99	13.02
ADM	1.60	0.96	12.97
STC1	3.13	1.41	12.62
PPP1R3B	1.70	1.17	12.57
CP	0.43	2.43	12.37
SPRY1	1.06	0.69	12.35
EFEMP2	1.53	1.07	12.01
TNS1	4.15	1.40	11.83
PFKFB4	2.57	2.17	11.60
BNIP3L	1.25	1.14	11.17
PPP1R3C	1.53	1.33	11.13
ENO2	2.58	0.95	10.43
PIK3IP1	1.21	1.43	10.09
TMEM158	2.27	1.66	9.85
TCP11L2	1.20	1.18	9.44
IGFBP3	1.83	1.19	9.20
FGF11	1.61	1.45	9.09
C7orf68	3.29	90.00	8.97
CITED2	1.15	0.85	8.86
STC2	1.65	6.54	8.58
KLHL24	1.04	0.80	8.58
FBXO32	1.49	2.02	8.47
KDM3A	1.33	1.27	8.41
PTPRB	1.18	1.31	8.33
ANKRD37	1.23	1.16	8.09
JHDM1D	1.20	1.03	7.56
TP3INP1	1.79	1.38	7.35
C5orf41	1.19	0.97	7.23
MT1X	1.33	1.14	7.06
HK2	1.96	1.96	6.97
SPAG4	2.27	1.29	6.77
FAM13A	1.63	1.78	6.71
P4HA1	0.98	0.79	6.55
MXI1	1.50	1.06	6.45
APOL2	8.50	14.50	6.42
ZNF292**	1.13	0.94	6.38

*RIMKLA was not one of the top50 genes with qualified H3K4me3 peaks in non-crosslinked chromatin because no peaks were identified within 5000 bp flanking the TSS.

**ZNF292 was the top 51 gene with qualified H3K4me3 peaks in crosslinked chromatin and the top 50 gene in non-crosslinked chromatin.

## Results

We previously reported that exposure of human lung adenocarcinoma A549 cell line to NiCl_2_ for 24 hr resulted in a 3-fold increase in global levels of trimethylated H3K4 as measured by Western blot analysis using an antibody specific for H3K4me3 [19]. In the present study, we further explore the genome-wide relationship between the levels and distribution of H3K4me3 by ChIP-Seq and the levels of gene expression by RNA-Seq induced by NiCl_2_. Total RNA was isolated from untreated A549 cells (control) or A549 cells treated with 1 mM NiCl_2_ for 24 hr and gene expression profiling was performed by RNA-Seq and nickel-induced genes were identified. Since increased levels of trimethylated H3K4 are associated with gene expression and mapped to promoter regions near TSSs, we selected genes with qualified H3K4me3 peaks within 5,000 bp upstream and downstream of TSS among the set of identified nickel-induced genes. The top 50 highest nickel-induced genes (genes with greatest fold-induction by nickel treatment identified by RNA-Seq) with qualified H3K4me3 peaks within 5,000 bp flanking both sides of the TSSs are listed in Table 2.

**Figure 1:**
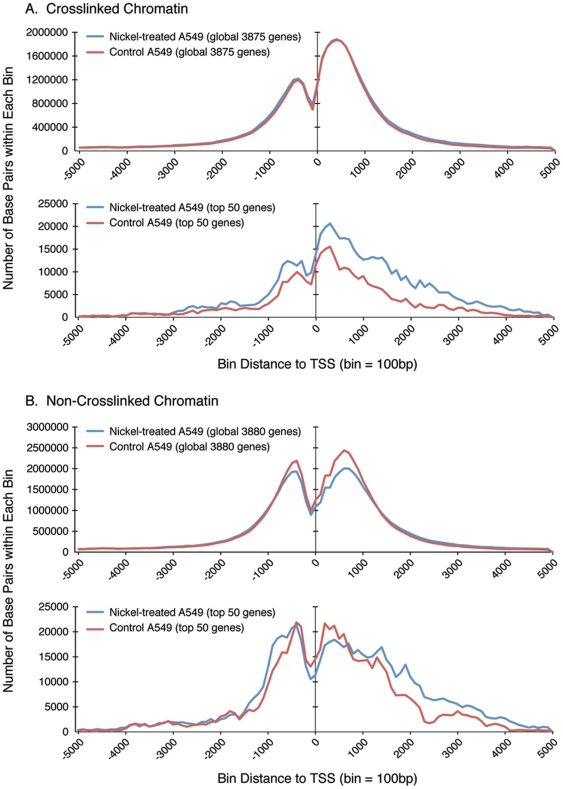
Global profiling of all nickel-induced genes and the top 50 highest nickel-induced genes with qualified H3K4me3 peaks within 5,000 bp upstream or downstream of TSS (center line at 0) isolated from (A) crosslinked chromatin and (B) non-crosslinked chromatin from untreated control (red) and nickel-treated (blue) A549 cells.

ChIP-Seq analysis was performed with an antibody specific for H3K4me3 on chromatin isolated from untreated and nickel-treated A549 cells after crosslinking with formaldehyde. H3K4me3 peaks were identified by comparison with input DNA, namely, chromatin isolated from control cells without antibody immunoprecipitation. As shown in Figure 1A (top panel), global H3K4me3 profiling of all 3,875 nickel-induced genes with qualified H3K4me3 peaks within 5,000 bp flanking the TSSs (center line at 0) isolated from crosslinked chromatin demonstrated two major peaks, a smaller pre-TSS peak and a larger post-TSS peak. After the pre-TSS peak, there was a significant dip in the signal at −100 (100 bp before TSS), followed by a post-TSS peak which peaked at +400 (400 bp after TSS). These findings are consistent with previous report of the loss of nucleosome and the assembly of the first nucleosome at TSS in active genes [19]. Interestingly, the global H3K4me3 profiles of all 3,875 genes in control (red) and nickel-treated (blue) A549 cells overlapped exactly. Next we examined H3K4 trimethylation coverage in the top 50 highest nickel-induced genes (top 50) (Table 2) in crosslinked chromatin isolated from A549 cells. In contrast to the global H3K4me3 profile of all 3,875 nickel-induced genes whereby the H3K4me3 peaks of untreated and nickel-treated cells overlapped, the pre-TSS and post-TSS peaks of the top 50 genes were higher in nickel-treated cells (blue) compared to control A549 cells (red) (compare Figure 1A, bottom panel with top panel). More interestingly, the post-TSS peak of nickel-treated cells remained higher than that of control cells over a broader region spanning over 4,000 bp downstream of TSSs (Figure 1A, bottom panel).

Since ChIP-Seq analysis has been performed using chromatin isolated from cells with or without formaldehyde crosslinking, we next examined H3K4me3 coverage in non-crosslinked chromatin isolated from untreated and nickel-treated A549 cells without formaldehyde crosslinking. H3K4me3 peaks were identified by comparison with similarly prepared input DNA without antibody immunoprecipitation. Global H3K4me3 profiling of all 3,880 nickel-induced genes with qualified H3K4me3 peaks within 5,000 bp flanking the TSSs isolated from non-crosslinked chromatin also demonstrated two major peaks spanning the TSSs (Figure 1B, top panel). In contrast to the smaller pre-TSS and larger post-TSS H3K4me3 peaks observed in crosslinked chromatin (Figure 1A), in non-crosslinked chromatin, the pre-TSS peaks were more similar in peak height with their respective post-TSS peaks for all 3,880 nickel-induced genes and the top 50 highest nickel-induced genes (Figure 1B, top and bottom panels). Surprisingly, in the absence of crosslinking, the post-TSS peak for control A549 cells was higher than that of nickel-treated cells globally for all 3,880 genes (Figure 1B, top panel). Similarly, the H3K4me3 peak for the top 50 genes in control cells was initially higher than that in nickel-treated cells up to +600 (600 bp after TSS) (Figure 1B, bottom panel). As the H3K4me3 profiling curve of control cells declined beyond the latter, the H3K4me3 profiling curve of nickel-treated A549 cells became higher than that of control cells.

**Figure 2:**
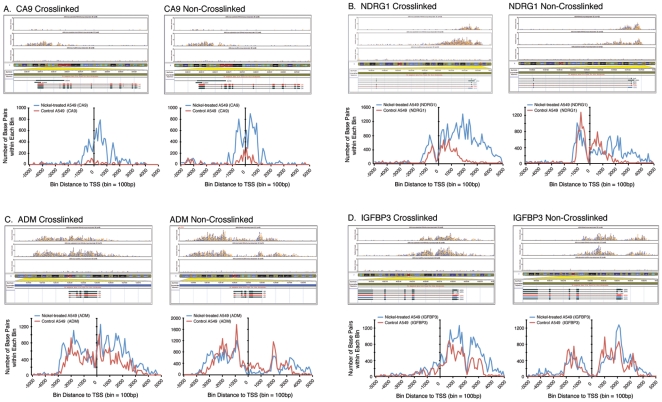
Comparison of H3K4me3 deposition in the promoter and coding regions of (A) CA9, (B) NDRG1, (C) ADM, and (D) IGFBP3 genes isolated from crosslinked or non-crosslinked chromatins from untreated, nickel-treated A549 cells and input DNA and aligned to the exons and introns of their respective genes (top panels). H3K4me3 profiling curves of the respective genes within 5,000 bp upstream or downstream of TSS (center line at 0) from crosslinked and non-crosslinked chromatins from untreated control (red) and nickel-treated (blue) A549 cells (bottom panels).

Because of the differences in the H3K4me3 profiles of the top 50 highest nickel-induced genes in crosslinked compared to non-crosslinked chromatins, we further examined the fold changes in H3K4me3 peaks for these genes. For genes with more than one qualified H3K4me3 peaks, only the peak with the highest fold change comparing nickel-treated versus control A549 cells was listed in Table 2. Although variations in fold change in H3K4me3 peaks were observed in crosslinked versus non-crosslinked chromatins for some genes, the same top 49 genes were identified to contain qualified H3K4me3 peaks within 5,000 bp flanking TSSs, irrespective of formaldehyde crosslinking. The H3K4me3 profiles of the two highest nickel-induced genes, namely, CA9 and NDRG1, from both crosslinked and non-crosslinked chromatins were further examined. The H3K4me3 profiling curves of the CA9 gene from both crosslinked and non-crosslinked chromatins demonstrated that nickel treatment increased both the pre-TSS and post-TSS peaks compared to control A549 cells (Figure 2A, bottom panels). For the NDRG1 gene, the post-TSS peak from crosslinked chromatin of nickel-treated A549 cells was significantly higher and broader, spanning up to 5,000 bp downstream of TSS, than that of the corresponding control cells and the post-TSS peak from non-crosslinked chromatin of nickel-treated A549 cells (Figure 2B, bottom panels). The H3K4me3 profiling curve of crosslinked NDRG1 gene after nickel treatment is consistent with the higher and broader H3K4me3 curve for the top 50 genes from crosslinked chromatin isolated from nickel-treated A549 cells (Figure 1A, bottom panel).

Upon closer examination of the H3K4me3 profiling curves for the rest of the genes in the top 50 gene list, an interesting H3K4me3-depleted or free region in non-crosslinked chromatin was discovered, most notably, in the ADM and IGFBP3 genes. In crosslinked chromatin for the ADM gene, there was a pre-TSS peak, followed by a dip right before TSS and then a region of ∼3,000 bp with H3K4 trimethylation after the TSS (Figure 2C, bottom panel). In contrast, in non-crosslinked chromatin, after the pre-TSS peak and the dip at TSS, H3K4me3 coverage significantly decreased in control cells and was absent in nickel-treated cells from the TSS at 0 to +1,800 before the reappearance of a sharp peak at +1,900. Similarly, in non-crosslinked chromatin of the IGFBP3 gene, after the pre-TSS peak, there was a region spanning from −200 to +400 totally free of H3K4 trimethylation in both control and nickel-treated cells, followed by the reappearance of H3K4me3 deposition at +600 (Figure 2D, bottom panel). The mechanism underlying the striking differences in H3K4me3 coverage in crosslinked versus non-crosslinked chromatins for the ADM and IGFBP3 genes remained to be determined.

**Figure 3:**
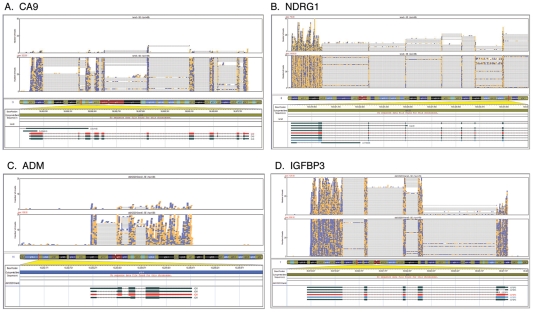
RNA-Seq analysis of the expression of (A) CA9, (B) NDRG1, (C) ADM, and (D) IGFBP3 genes induced by nickel treatment (lower panel) compared to untreated control (upper panel) in A549 cells and aligned to the exons and introns of their respective genes.

**Figure 4:**
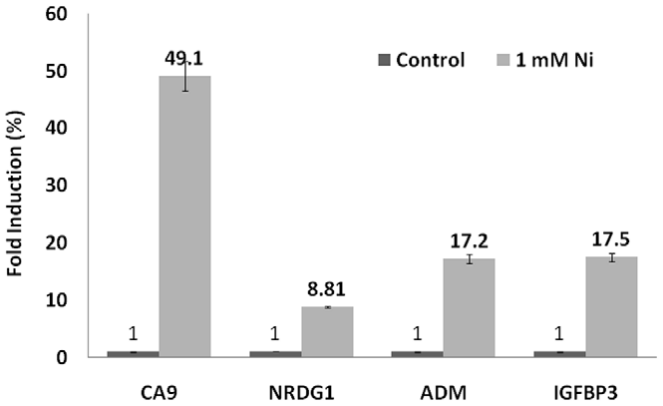
Real Time-PCR (RT-PCR) analysis of the relative level of induction (fold induction depicted as %) of expression of CA9, NDRG1, ADM, and IGFBP3 genes in nickel-treated A549 cells compared to untreated control cells.

Next, the induction of expression of CA9, NDRG1, ADM and IGFBP3 genes by nickel treatment in A549 cells was evaluated by RNA-Seq and aligned to the exons and introns of their respective genes (Figure 3, A–D). The fold induction of expression of these genes by nickel treatment as determined by RNA-Seq was listed in Table 2. The level of induction of expression of CA9, NDRG1, ADM and IGFBP3 genes by nickel treatment was further confirmed by Real Time-PCR (RT-PCR) analysis and shown in Figure 4.

**Figure 5:**
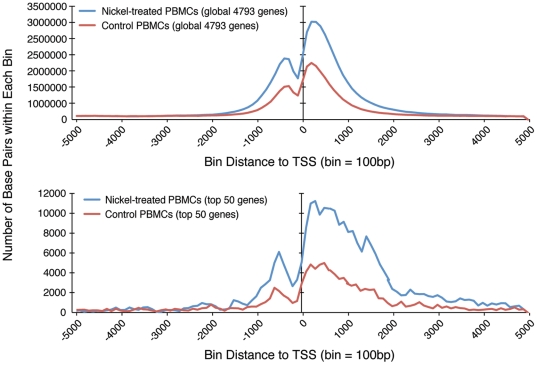
Global profiling of all nickel-induced genes and the top 50 highest nickel-induced genes with qualified H3K4me3 peaks within 5,000 bp upstream or downstream of TSS (center line at 0) isolated from crosslinked chromatin from untreated (red) and nickel-treated (blue) peripheral blood mononuclear cells (PBMCs).

**Figure 6:**
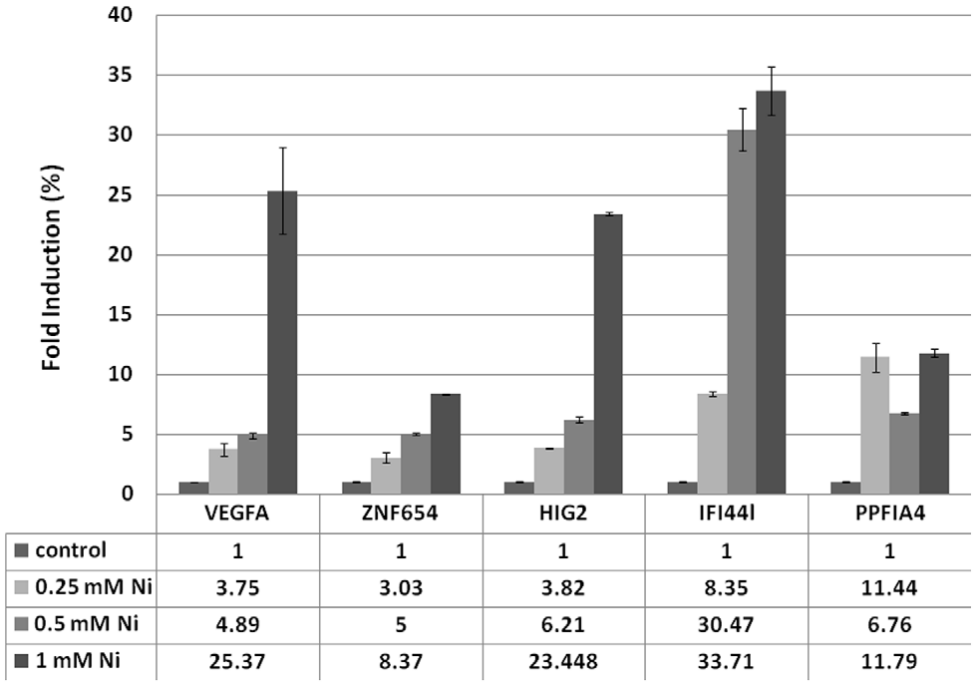
Real Time-PCR analysis of the relative level of induction (fold induction depicted as %) of expression of VEGFA, ZNF654, HIG2, IFI44L and PPFIA4 genes in nickel-treated PBMCs compared to untreated control cells.

To further examine if the effects of nickel treatment in increasing the height and broadening of the H3K4me3 peaks observed in the top 50 highest nickel-induced genes from crosslinked chromatin from A549 cells (Figure 1A, bottom panel) can be observed in another cell type, peripheral blood mononuclear cells (PBMCs) were isolated and treated with 0.5 mM NiCl_2_ for 24 hr or untreated as control. Cells were crosslinked with formaldehyde before isolation of chromatin and H3K4me3 ChIP-Seq analysis was performed. As shown in Figure 5, global H3K4me3 profiling of all 4,793 genes in PBMCs with H3K4me3 peaks within 5,000 bp flanking the TSSs demonstrated two major peaks, a smaller pre-TSS peak and a larger post-TSS peak, similar to that observed in crosslinked A549 cells (Figure 1A). Interestingly, nickel treatment increased the height of the H3K4me3 peaks in addition to broadening of the H3K4me3 peaks in not only the top 50 highest nickel-induced genes but also globally in all 4,793 genes (Figure 5). The level of induction of expression of five nickel-induced genes, namely, VEGFA, ZNF654, HIG2, IFI44L and PPFIA4, was confirmed by RT-PCR analysis and shown in Figure 6. Interestingly, nickel treatment induced the expression of ADM but not CA9, NDRG1 and IGFBP3 in PBMCs.

## Discussion

Although, epidemiological, animal, and cell culture studies have found nickel compounds to be carcinogenic [14], [25]–[27], the precise mechanism(s) of nickel carcinogenesis is not well understood. The carcinogenic properties of nickel may be attributable in part, to activation and/or repression of gene expression induced by changes in the DNA methylation status and histone tail post-translational modifications. Previous work from our group has shown that exposure of cells to nickel alters histone post-translational modifications. For example, nickel increased H3K9 dimethylation by inhibiting the demethylating enzyme JMJD1A [28], [29] suggesting that the carcinogenic effects of nickel may involve aberrant changes in gene expression through epigenetic mechanism(s). We recently reported that hypoxia increased the levels of H3K4me3 by inhibition of JARID1A demethylase [30]. The induction of hypoxia signaling by exposure to NiCl_2_ also significantly increased the levels of H3K4me3 after 24 hr or 7 days of exposure in the human lung carcinoma A549 cells [19], supporting a plausible mechanism of carcinogenicity of nickel that involves the induction of H3K4 trimethylation and expression of genes that may cause cell transformation. Since nickel is a hypoxia mimetic, many nickel-induced genes are also induced by hypoxia, including CA9 and NDRG1, the two highest nickel-induced genes in A549 cells (Table 2). Acidosis of the tumor microenvironment is common in hypoxic tumors which results in the induction of CA9. CA9 catalyzes the reversible hydration of carbon dioxide into bicarbonate and protons, thereby contributing to the maintenance of a more alkaline intracellular pH and tumor survival [31]. NDRG1 is expressed at low levels in normal tissues but its expression is increased in a variety of cancers, making it an important cancer marker. NDRG1 is both hypoxia and Ca^2+-^responsive and its induction may be HIF-1-dependent or independent [32]. ADM is also a hypoxia-inducible gene which plays an important role in tumor progression and angiogenesis while IGFBP3 is a pro-apoptotic gene induced by hypoxia [33], [34]. Further understanding of the induction of H3K4 trimethylation and increased expression of these genes will provide better insight into the epigenetic effects underlying the carcinogenicity of nickel compounds.

Since H3K4 trimethylation at the promoter region of genes has been linked to transcriptional activation, we performed genome-wide mapping of H3K4me3 deposition in the promoter regions within 5,000 bp flanking the TSSs by ChIP-Seq and matched with genes identified by genome-wide mapping of RNA transcripts by RNA-seq. The combination of ChIP-Seq and RNA-Seq provides a robust approach for the interrogation of the involvement of the activating mark H3K4me3 and its distribution in genes induced after exposure to nickel.

Consistent with the induction of gene expression observed after nickel treatment, the post-TSS H3K4me3 peak of the top 50 highest nickel-induced genes isolated from crosslinked chromatin was higher and broader after nickel treatment compared to untreated control (Figure 1A, bottom panel). In contrast, when the chromatin was not crosslinked with formaldehyde, both the control and nickel-treated H3K4me3 peaks were similar in height and were broader in the top 50 highest nickel-induced genes (Figure 1B, bottom panel). These findings prompted further investigation of H3K4me3 coverage at the level of individual genes.

As shown in Figure 2A, nickel induced higher H3K4me3 peaks in the CA9 gene in both crosslinked and non-crosslinked chromatins. In the NDRG1 gene, the post-TSS H3K4me3 peak induced by nickel was both higher and broader spanning up to 5,000 bp after the TSS in crosslinked chromatin compared to non-crosslinked chromatin (Figure 2B). Interestingly, for other nickel-induced genes such as ADM and IGFBP3, the distribution of H3K4me3 in the promoter region and along the gene body was strikingly different between crosslinked and non-crosslinked chromatins. Most notably, the robust H3K4me3 deposition immediately downstream of the TSS in the ADM gene that spanned from the TSS up to the first four exons of the gene in crosslinked chromatin was completely absent in non-crosslinked chromatin (Figure 2C). Similarly, H3K4me3 deposition was observed in the first exon of the IGFBP3 gene immediately downstream of TSS in crosslinked chromatin but not in non-crosslinked chromatin (Figure 2D).

At the present time, we cannot explain the loss of H3K4me3 deposition at the ADM and IGFBP3 genes only in non-crosslinked but not crosslinked chromatins. We can only speculate that the difference between crosslinked and non-crosslinked chromatins is that crosslinking by formaldehyde represented a “snapshot” of the equilibrium of the nucleosome occupancy and H3K4me3 distribution along the genes at the time of crosslinking [35]. In contrast, during the time period of harvesting of chromatin without formaldehyde crosslinking at 4°C, the dynamic process of H3K4 methylation and/or distribution can continue to occur in intact cells until the cells are finally lyzed. The loss of H3K4me3 coverage can be explained by the continued activity of demethylase that removes the methylated group from H3K4me3 in non-crosslinked chromatin during the time period of cell harvesting. Alternatively, the loss of H3K4me3 coverage can also be explained by the dynamic process of chromatin remodeling including nucleosome sliding and ejection [36] which may continue to occur until cells are lyzed. Hence, in non-crosslinked chromatin, it is plausible that nucleosomes containing H3K4 trimethylation can slide down the gene body further downstream from the TSS or can be ejected from the regions immediately downstream of TSSs, leaving the 5′end of the ADM and IGFBP3 genes deficient in the H3K4me3 activating mark. Although the mechanism underlying the loss of H3K4 trimethylation remains to be investigated, our results demonstrated that comparison of the distribution of histone marks in crosslinked and non-crosslinked chromatins may potentially offer new insights into the dynamic process of nucleosome remodeling and histone modifications.

The effect of nickel on H3K4 trimethylation is considerably greater than can be assessed using Chip-Seq. The reason for this is because equal amounts of immunoprecipitated DNA were sequenced in untreated and nickel-treated cells, yet there was a 3-fold increase in DNA precipitated with the H3K4 trimethylation antibody from nickel-treated cells. Thus the effects of nickel exposure on H3K4 trimethylation are considerably greater than portrayed in our results. Our results also point to the importance of using formaldehyde crosslinking to capture the “snapshot” of the equilibrium of the changes in H3K4 trimethylation induced after nickel exposure. Previous studies using Chip-Seq to map histone modifications did not use formaldehyde crosslinking. However, these investigators were not trying to measure an effect of treatment with an agent on histone modifications [13]. On the other hand, the mapping of histone modifications in the absence of formaldehyde crosslinking offers an opportunity to investigate the dynamic processes of nucleosome remodeling and histone modifications. Whereas previous studies have localized H3K4 trimethylation to the promoter of genes, our results indicate, especially following nickel treatment, that this modification can extend great distances into the coding region of genes that are induced after nickel exposure.
